# The emotions experienced by family medicine residents and interns during their clinical trainings: a qualitative study

**DOI:** 10.1017/S1463423623000051

**Published:** 2023-04-05

**Authors:** Ozlem Tanriover, Sukran Peker, Seyhan Hidiroglu, Dilek Kitapcioglu, Sinem Yildiz Inanici, Nesrin Karamustafalioglu, M. Ali Gulpinar

**Affiliations:** 1 Department of Family Medicine and Medical Education, Faculty of Medicine, Yeditepe University, Istanbul, Turkey; 2 Department of Public Health, Marmara University School of Medicine, Istanbul, Turkey; 3 Department of Medical Education, Acıbadem Mehmet Ali Aydinlar University, Istanbul, Turkey; 4 Department of Medical Education, Marmara University School of Medicine, Istanbul, Turkey; 5 Department of Psychiatry Bakirkoy Research and Training Hospital for Psychiatry, Neurology and Neurosurgery, Istanbul, Turkey

**Keywords:** doctor–patient communication, emotions, family medicine residents, feelings, interns, medical students

## Abstract

**Background:**

The family medicine residents and final year medical students are challenged with increased workload and they experience various emotions during their clinical trainings. They are confronted with uncertainties in their role descriptions and they witness illness, suffering and deaths as part of their everyday duties which may lead to burnout. Only several studies have focused on these experiences to find out what the family medicine residents and medical students were literally feeling.

**Aim:**

The aim of this study was to explore the family medicine residents’ and final year medical students’ emotions during their clinical trainings.

**Method:**

This qualitative study was performed with 15 family medicine residents and 24 final-year medical students using a convenience sample from two medical faculties to explore and analyze their emotions. Data were gathered by means of focus group interviews, including six interviews conducted and recorded through online meetings. Data were analyzed for themes using a thematic analysis approach. Since the interviews reached saturation in terms of content, the interviews were terminated at the end of sixth focus group meetings. Each interview took an average of 45–60 min.

**Results:**

Three main themes emerged from the data regarding residents’ and interns’ emotions. These were the “clinical climate’s role”, “emotions during patient encounters” and “coping strategies with negative emotions”. The most commonly encountered emotions were tension and anxiety followed by frustration and uncertainty.

**Conclusions:**

The family medicine residents and final-year medical students are challenged with emotions during their clinical trainings. Therefore, medical educators have to be aware of the need to support them in reflecting their emotions by prioritizing residents’and interns’ well-being.

## Introduction

Medical education is typically regarded as highly stressful (Ofri, 2013). Doctors are trained in a culture that has high expectations. High achievers compete with each other with little or no room for making an error in order to strive for excellence. Moreover, medical students or the residents are confronted with uncertainties in their role descriptions, and they witness illness, sufferings and deaths as part of their everyday duties. Both medical students and family medicine residents work under pressure which may easily lead to burnout (Soler *et al.*, 2008). In an European General Practice Research Network (EGPRN) study including 12 European countries (*n* = 1393) in terms of burnout, 43% of respondents scored high for “emotional exhaustion burnout”, 35% for “depersonalization” and 32% for “personal accomplishment”, with 12% scoring high burnout in all three dimensions (Soler *et al.*, 2008). In a Turkish study, Kosan *et al* reported about 70% of burnout among family physicians (*n* = 246). Their study exhibited a higher level of “emotional exhaustion” (Kosan *et al.*, 2019).

During this heavy workload, medical students or residents do not have time to think, talk or reflect on their emotions (Helmich *et al.*, 2011a). In fact, being aware of and able to regulate emotions is essential to doctor–patient relationship and to build medical teamwork. Furthermore, being aware of and able to understand and manage emotions in oneself and others is critical for medical students’ and family medicine residents’ personal wellbeing (Satterfield and Hughes, 2007; Shapiro, 2011).

Medical students and family medicine residents may face intense emotions in patients causing similar emotional reactions in themselves (Helmich *et al.*, 2011b). However, evidence shows that emotional learning processes tend to be underestimated (Karnieli-Miller *et al.*, 2010), and doctors may seem reluctant to confront their own emotions (Helmich *et al.*, 2011a; 2011b). Being able to understand and regulate emotions is considered a critical feature of medical students’ and residents’ overall clinical performance, including diagnostic processes, medical decision-making, and interpersonal relationships (Croskerry *et al.*, 2008). However, only a few publications demonstrated that there was a need for a set of skills that medical students or residents should develop in dealing with emotions (Satterfield and Hughes, 2007; Shapiro, 2011; Cherry *et al.*, 2012).

Therefore, the aim of this study was to explore the family medicine residents’ and final-year medical students’ emotions during their clinical trainings. In addition to that we wanted to find out the emotions experienced during their patient encounters and the strategies they use to regulate emotional experiences and responses to stress.

## Method

This qualitative study was performed with 15 family medicine residents and 24 final-year medical students using a convenience sample from two medical faculties to explore and analyze their emotions during their clinical trainings.

Data were gathered by means of focus group interviews, including six interviews conducted and recorded through online meetings.

An information meeting on the subject was held for the residents and students, and volunteers were included in the study.

Interns and residents who agreed to participate in the study were invited to the online platform where the interview would take place. The meeting was held with a facilitator and an observer. At the beginning of the interview, the participants were informed about the subject and purpose of the study. In addition to that, their verbal consent for the study and recordings was obtained. The interviews were recorded both as audio-video and by observer notes. Sessions lasted approximately 45 min to 1 h.

The interviews were conducted by three researchers (OT, SP, SH) who were trained in qualitative research.

Participants were asked about demographic characteristics in the first part of the interview, and then semi-structured questions created by literature review were asked to the participants.

The main questions included in focus groups were:

1. What kind of emotions in general do you experience in the clinical settings?
2. What kind of emotions do you experience while having a patient–physician interviews?
3. How have you been able to cope with emotionally difficult situations?

A focus group format was selected for this study, as this method is useful for exploring views, opinions, knowledge, experiences and needs of participants.

With relatively few qualitative studies on the topic, an inductive thematic analysis approach was selected for this exploratory study. A preconceived theoretical framework was not used; instead, the researchers allowed themes to emerge as the data were analyzed.

## Data analysis

Data were analyzed by using a “thematic analysis” approach. In this analysis, we used the six steps proposed by Braun and Clarke (Braun and Clarke, 2006).

During the initial step, two researchers reviewed the transcripts to better understand the content. In the second step, the primary researcher labeled the important issues of the data. After that, the codes were labeled based on the interpretations using open coding. Transcripts were independently read and coded by the two authors. In the third step, relationships among the concepts were investigated and emerged into themes. In the fourth step, discussion and comparison of coding led to the identification of themes. In the fifth step, each theme was defined and named. Finally, in the sixth step, the relationships between the themes were discussed and a report was written. This was reviewed by the research team and revised through discussion. A final code book was agreed upon that included clear definitions of themes and sub-themes.

Since the interviews reached saturation in terms of content, the interviews were terminated at the end of sixth focus group meetings. Each interview took an average of 45–60 min.

Six video recordings were performed with the permission of the participants during the interviews.

The audio recordings of the interviews were transcribed by the researchers who conducted the interview on the same day.

## Ethics committee approval

This study was approved by the local Research Ethics Board.

Permission was obtained from the ethics committee for video recordings as well.

The informed consent form of the study was verbally explained to the participants and their verbal consent was obtained for the study and video recordings. All data were analyzed anonymously. Our research was prepared in accordance with the Declaration of Helsinki, which was revised in 2000.

## Results

A total of 39 participants were interviewed. Of these, 26 were female. The mean age was 25.41 ± 1.72 (min 23–max 32). Fifteen were second year family medicine residents and had experience of a GP placement in their rotations. Fourteen were final year medical students (interns) from a governmental medical school while 10 were interns from a foundation university. Demographic details were shown in Table 1.

**Table 1.** Information of the participants in the research (*n* = 39)

Participants	Number	Number of focus groups	Sex F/M	Age
FM Residents	15	2	9 /6	25–32
Governmental University Interns (final year medical students)	14	2	10 /4	24–30
Foundation University Interns (final year medical students)	10	2	7 /3	23–27
Total	39	6	26/13	The mean age: 25.41 ± 1.72

FM = family medicine.

The interns and residents were happy to share their emotions frankly and were grateful to the researchers as no one else has asked them about their feelings before.

Themes and subthemes are described below, with participant quotations identified by participant’s gender, profession (intern or resident), faculty (Foundation University: FU; Governmental University: GU) and age which was given in the parenthesis (Table 2).

**Table 2.** Three main themes and their subthemes using thematic analysis methodology

Main Themes	Subthemes	Emotion labels related to subthemes	Sample Participant Quote
“Clinical climate’s role”	1. “feelings of being unfamiliar” 2. “feelings of not being understood or valued” 3. “communication problems due to uncertain role descriptions”	feelings of worthlessness, helplessness, tension and anxiety followed by frustration and uncertainty.	*‘…again, I found myself alone dealing with this acute medical emergency no one else is helping me in the clinic.’ (M, Resident, M, 25y).*
“Emotions during patient encounters”	1. “feelings depending on patients’ condition” 2. “hiding emotions during patient encounters”	“excitement, stress, feelings of insufficiency, inadequacy, feelings of anger towards patient or loosing calmness, uncertainity, happiness, compassion, relief, joy, pride, satisfaction and confidence followed by burnout/exhaustion, boredom, upset, sadness, anger and anxiety and finally loss of feelings”	*“….Besides, if the patient is a complicated case, I feel stressed and I have thoughts like what would I do if I were alone, could I be enough?” (F, Intern, Y, 24y)*
“Coping strategies with negative emotions”	1. “emotional awareness” 2. “accepting the situation” 3. “loss of feelings”	“calming down, feeling of acceptance, and hope for the future, behaving like a robot”	*“…examining too many patients makes you numb-you become like automated”(M, Resident, F,27 y).*

**Figure 1.**
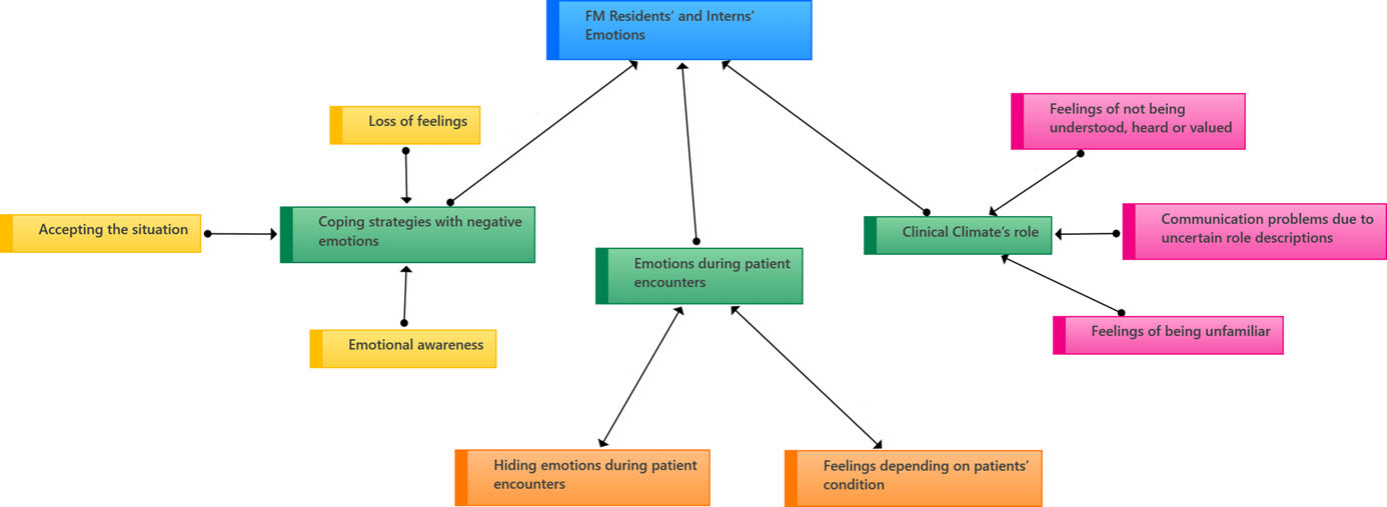
Concept map displaying the assocaitions among the themes and subthemes related to emotions experienced during clinical trainings.

Overall three main themes emerged from our data regarding residents’ and interns’ emotions. These were the “clinical climate’s role”, “emotions during patient encounters” and “coping strategies with negative emotions”. The main themes and the subthemes are demonstrated in a concept map in Figure1.

## “Clinical climate’s role”

Our first theme was the “**clinical climate’s role**”. When interns or residents start a new rotation, they are unfamiliar with this new environment and if there is no one else to do an orientation or if the medical staff (nurses, residents. etc) were too busy and seem to be not very helpful, the newcomers may feel like they are not welcomed to this clinic and state that they might have negative feelings onward. Moreover, they also state that in some clinics they are ignored or not heard or valued. Therefore, the subthemes associated with our first theme were as the students/residents not being used to the routines of the clinic they experienced “feelings of being unfamiliar”, “feelings of not being understood, heard or valued” and “communication problems due to uncertain role descriptions” all of which can prevent the achievement of the goals of the clinical education. All of these experiences can have some negative effects on the students’ thought content or emotional world. The emotion labels related to these subthemes were the feelings of worthlessness, helplessness, tension and anxiety followed by frustration and uncertainty. Below are some of the examples related to this theme:

*“….in the clinic I’m sick of trying to be useful, what do I do to the nurse? …she doesn’t record or doesn’t want to understand, even if I express it as accurately as I can. I don’t feel understood, I feel like worthless, feel very helpless” (F, Intern, FU, 24 y)*.

*‘…again, I found myself alone dealing with this acute medical emergency no one else is helping me in the clinic.’ (M, Resident, GU, 25y)*.

In Turkey, final-year medical students are called “intern doctors,” and they are no longer students, but on the other hand, they are not graduated as medical doctors. For this reason, they express that they have problems with other healthcare team members because of this uncertainty. Interns also stated that they had difficulties in the clinic, in relations with patients, their relatives, relations with other healthcare professionals, and in recognizing their own roles and responsibilities. Interns stated that defining their roles in the clinical environment is important not only for themselves, but also for other healthcare professionals, patients and educators, and they have difficulties in the clinical settings when the roles are not defined well.

Below is an example related to this subtheme:

*“Different attitudes created confusion about what we should do and should not do. They did not allow us to do anything when we were interested. When we kept our distance and stand on the sidelines, we were pressured to answer why we weren’t interested this time” (M, Intern, FU, 25y)*.

## “Emotions during patient encounters”

Our second theme was “**emotions during patient encounters**”. Under this theme, we have identified two subthemes: “**feelings depending on patients’ condition**”and “**hiding emotions during patient encounters**”.

### “Feelings depending on patients’ condition”

Both residents and interns stated that their feelings changed according to patients’ condition. If they find the patient to be too complex then they had feelings of insufficiency. They thought that they had insufficient medical knowledge and skills. They feel uncertain in their medical knowledge and skills and they have the fear of making mistakes followed by stress and anxiety:

*“….Besides, if the patient is a complicated case, I feel stressed and I have thoughts like what would I do if I were alone, could I be enough?” (F, Intern, FU, 24y)*

*“… during our medical education we memorize the rarest things, syndromes etc. But when it comes to patient encounter, I can’t help, but, feel stressed and inadequate….” (F, Resident, GU, 29y)*

On the other hand if the chief complaint seemed too minor, then they express feelings of anger toward patient or loosing calmness:

*“….in the ER, in the middle of the night that man comes with first degree burn in his hand. I really get mad, because he is stealing my time and he is stealing from other patients’ time…”(M, Resident, GU, 29y)*

If the residents or the interns think that they have managed the patient well, then they have the feelings of happiness, compassion, relief, joy, pride, satisfaction and confidence:

*“…It’s a relief when you examine and treat such a patient. I feel joy, happiness and satisfaction after seeing the patient…”(M, Intern, FU, 24y)*.

### “Hiding emotions during patient encounters”

Another subtheme was “**hiding emotions during patient encounters**”. Both residents and interns stated that they struggled with hiding their emotions from the patients. The majority of interns and residents believed that physicians should hide their emotions from their patients and that showing the emotions openly was unprofessional. Furthermore, they thought that becoming more apathetic and to be able to distance themselves from the patients will be an asset for them in their medical career.

Although they experienced positive emotions such as excitement, joy, pride and happiness, they thought that they should not share these feelings with their patients. They also stated that they should hide their negative emotions like burnout/exhaustion, boredom, stress, upset, sadness, anger and anxiety. Below are some examples:

*“…when physicians make decisions that will affect the life of that patient, only science can influence medicine, we must act without causing emotionality” (M, Resident, GU, 25 y)*.

*“…even if we are doctors, we are all human, we have feelings. But when we are seeing the patient that sentimentality should stay out of the door” (F, Intern, FU, 23y)*.

The emotion labels related to these subthemes under the theme of “**emotions during patient encounters**”were the “excitement, stress, feelings of insufficiency, inadequacy, feelings of anger toward patient or loosing calmness, uncertainity, and feelings of happiness, compassion, relief, joy, pride, satisfaction and confidence followed by burnout/exhaustion, boredom, upset, sadness, anger and anxiety.

## “Coping strategies with negative emotions”

Our third theme was “**coping strategies with negative emotions**”. It is seen that residents or the interns cope with emotions in various ways. Under this theme, we have identified three subthemes: **“emotional awareness”, “accepting the situation**” and **“loss of feelings”.**

### “Emotional awareness”

Residents and interns mentioned the importance of awareness of their own emotions and expressing their feelings was the first step in improving coping strategies and better clinical outcomes. Below are the statements of the residents and interns regarding this theme:

*“I know that I need to calm my current emotional intensity, calm it down. After I calm down, I communicate with the patient. If I don’t communicate with the person I’m having trouble with, I can’t relax. This is how I deal with my problems…” ”(F, Intern, FU, 24 y)*.

*“It’s like being… I’m trying to get over it this way. I use positive language to inspire myself. I have difficulties with words while emphasizing the importance of positive language for coping”*

*(F, Resident, GU,32 y)*

### “Accepting the situation”

On the other hand interns and residents cope with their emotions reminding themselves that emotionally intense situations will be part of their job and they need to get used to it.

*“…I try to adapt and accept the problems I face, thinking of the worst. When my patient died, I could not cope. Every time I go to the hospital I wondered if my patient will die today too? I will lose another patient every week I come. ….but I don’t know how it happened, frankly, …then maybe I accepted. This is my profession, I am a healthcare worker and it is in the nature of my profession to lose people as well as to win. Knowing this, I must continue my profession”(F, Intern, FU,26 y)*.

*“I started to think that the feeling of acceptance begins with the expression of your own emotions first. …I don’t compare myself to anyone. I accept this way. It’s your problem…I mean something you can deal with. Maybe I need time. Maybe I need another feeling. Hope or motivation” (F, Resident, GU,,27 y)*.

The emotion labels related to these subthemes under the theme of “Coping strategies with negative emotions” were the “calming down, feeling of acceptance, and hope for the future”.

### “Loss of feelings”

Our third subtheme was the “**loss of feelings**”. Seeing too many patients and trying to hide emotions may have caused the loss of feelings. In addition to that losing feelings could be related with denial of situations in the experience of patient encounters. Residents and interns described this situation as “behaving like a robot”. They think that they distance themselves from the patient’s feelings in order to protect themselves from distress:

*“In order to be able to do this profession, I say to myself: You are a doctor and everyone has a duty, a purpose for existence. That patient…she needs you and you should do your best for her well-being. You cannot feel sorry for her” (M, Intern, FU, 24y)*.

*“As I am still a student, I feel a slight excitement and also stress. Being a doctor is like a role played most of the time, and we all play the subconscious doctor type with our own language. I can also connect it to some kind of an auto-pilot” (M, Intern, FU, 23y)*.

*“…examining too many patients makes you numb-you became like an automated robot”(M, Resident, GU,27 y)*.

## Word cloud

**Figure 2.**
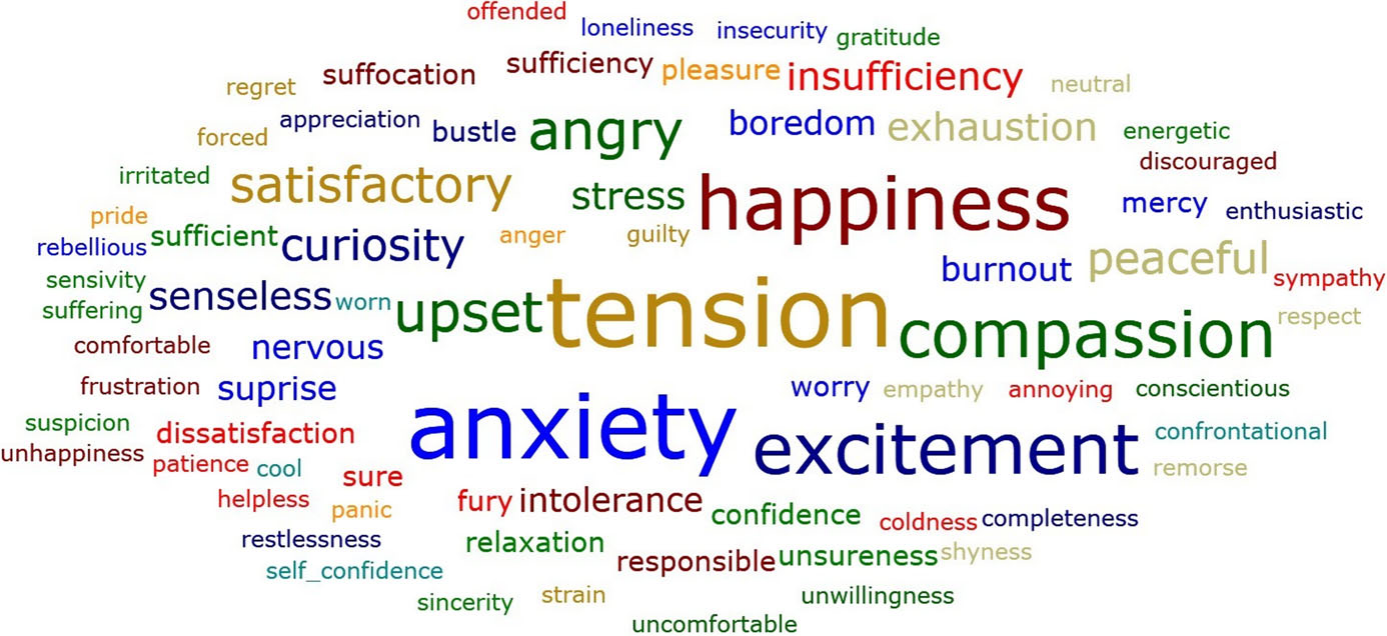
Word cloud of the emotions experienced by residents and medical students displayed according to word frequency.

We have shown the overall emotions in “Word Cloud” in Figure 2. The most commonly perceived emotions were the feelings of “tension and anxiety” followed by “happiness, compassion and excitement”. In addition to that “burnout, exhaustion, boredom, stress, angry and upset” were also frequently mentioned feelings.

## Discussion

To our best knowledge, this is the first study to explore the emotions of family medicine residents and interns during their clinical trainings in Turkey.

During clinical trainings, both family medicine residents’ and interns’ emotional experiences are critical for several reasons. First of all, emotions have important implications on cognitive processes such as learning and motivation (Pekrun *et al.*, 2002). Secondly, emotions help residents’ or interns’ professional identity development (Helmich *et al.*, 2012; Helmich *et al.*, 2014; Dornan *et al.*, 2015). Thirdly, during challenging conditions, high stress and anxiety may cause decline in empathy, blunting of emotions, and consequently may lead to burnout (Lee *et al.*, 2008b; Soler *et al.*, 2008; Paro *et al.*, 2014; Romani and Ashkar, 2014).

Therefore, to be aware of one’s own emotions can help to cope with emotional reactions. By this skill, residents or interns can promote their professional well-being. Because of this, there is a need for identifying and reflecting on emotional experiences in medical education (Post, 1997b; Thommasen *et al.*, 2001b).

In this study, our first theme was the “clinical climate’s role”. In Turkey, the family medicine residents and the interns have to do their core rotations that may last up to 4 months or longer at the hospital. During these rotations, the family medicine residents and the interns have the similar responsibilities as the residents of that clinic. Sometimes it is perceived that rotationers will come and go, therefore, there may not be an accommodating climate in that particular clinic.

The subthemes associated with our first theme were; as the interns/residents were not being used to the routines of the new clinic they experienced “feelings of being unfamiliar”, “feelings of not being understood, heard or valued” and “communication problems due to uncertain role descriptions” all of which can prevent the achievement of the goals of the clinical education. Medical education programmes are designed as a rotation through a series of departments at regular intervals. When residents or interns start a new rotation, they need supervision and support to adapt to a new clinical settings (Holmboe *et al.*, 2011). Therefore, in order to overcome feelings of unfamiliarity, when a resident or intern start a new rotation there should be orientation sessions. Many residents find transition experiences difficult and stressful (Brennan *et al.*, 2010; Sturman *et al.*, 2017; Coakley *et al.*, 2019).

Support and positive climate created by clinical consultants may decrease the initial stressors for trainees (Wiese and Bennett, 2022).

In addition to this, in this study, we have demonstrated that family medicine residents and interns struggle with emotional strains during their clinical trainings and patient–doctor interviews. The family medicine residents and interns mentioned feeling various emotions as well as blunting of the feelings in their descriptions that formed our three main themes. These were the “clinical climate’s role”, “emotions during patient encounters” and “coping strategies with negative emotions”. The most commonly perceived emotions were the feelings of “tension and anxiety” followed by “happiness, compassion and excitement”. In addition to that “feeling burnout, exhaustion, boredom, stress, angry and upset” were also frequently mentioned emotions.

Several studies show that medical students experience intense emotions during their patient encounters (Pitkälä and Mäntyranta, 2004).

For example Clay *et al* described themes of emotions as follows: sorrow, gratitude, personal responsibility, regret, shattered expectations and anger (Clay *et al.*, 2015). Other studies mainly focusing on empathy reveal the feelings of uncertainty and helplessness.

(Halpern, 2003; Nevalainen *et al.*, 2010; Neumann *et al.*, 2011; Burks and Kobus, 2012; Nevalainen *et al.*, 2012; Preusche and Lamm, 2016).

In addition to this, in an interprofessional study, the emotions of anxiety, sadness, empathy, frustration and insecurity were reported during difficult healthcare conversations (Martin Jr *et al.*, 2015). Besides several studies, the emotions of interns or the family medicine residents have rarely been the focus of systematic research. In the literature, typically the students’ or the residents’ intense emotions were the focus of attention in challenging situations such as anatomy dissections, autopsy encounters, or in case of a patient death (Bamber *et al.*, 2014; Sándor *et al.*, 2015; Trivate *et al.*, 2019).

Other than these highly emotional situations, the emotions of medical students or residents were not the leading actor (main character) in the clinical research scene. Therefore, researchers of the current study believe that interns or residents were grateful to the researchers for letting them express their emotions is also a finding and shows that there is a critical need for reflection/mindfulness sessions for residents and interns to learn how to be aware of their emotions and then how to express and regulate their emotions during their clinical trainings.

Besides intense emotions encountered during anatomy dissections, autopsy sessions or in case of a patient death, it has been stated that patient encounters and communication practices, which play a major role in the development of professional identity, are also emotionally challenging and stressful for students or residents (Sharif and Masoumi, 2005; Arieli, 2013).

In our study, both residents and interns have revealed that their feelings changed according to patients’ condition. If they find the patient to be too complex then they had feelings of insufficiency. They thought that they had insufficient medical knowledge and skills. They feel uncertain in their medical knowledge and skills and they have the fear of making mistakes followed by stress and anxiety. When it is extreme or prolonged, stress can create several health problems including burnout. Physical and mental health problems may appear due to burnout and may have direct effects on the quality of care provided to patients. Stress in the workplace has been identified as a major problem for family physicians (Post, 1997a). The term burn-out is defined as a combination of emotional exhaustion, feelings of depersonalization and perceived lack of personal accomplishment. A survey of rural family physicians in 2001 showed a self-reported burnout rate of as high as 55% (Thommasen *et al.*, 2001a). It is known that burnout rates increase during residency; therefore, interventions in medical education are necessary to identify the emotions that residents or interns may feel and cope with (Soler *et al.*, 2008; Lee *et al.*, 2008a; Romani and Ashkar, 2014).

Reflections are needed to normalize their emotions. This may allow residents or interns to develop a resilience to emotionally challenging situations.

On the other hand, if the residents or the interns think that they have managed the patient well, then they have the feelings of happiness, compassion, relief, joy, pride, satisfaction and confidence. On the contrary, if the chief complaint seemed too minor, especially during ER visits, then the residents and interns express feelings of anger toward patients or mentioned about losing their temper. Similarly, Isbell *et al*, using grounded theory, conducted 86 semi-structured qualitative interviews with experienced emergency department (ED) providers. They found out that patients triggered both positive and negative emotions. In their study, providers described feelings of frustrations with certain types of ED visits, which were inappropriate especially for services that are unnecessary for the ED to provide (eg, treatment for seasonal colds) (Isbell *et al.*, 2020).

Our final theme was “coping strategies with negative emotions”. It is seen that residents or the interns cope with their emotions in various ways. Under this theme we have identified three subthemes: “emotional awareness”, “accepting the situation” and “loss of feelings”.

Self-awareness is the ability to know one’s emotions, strengths and weaknesses, and it is one of the vital components of emotional intelligence. It is the ability to be aware of and to understand emotional states in oneself and others and to regulate one’s emotions effectively. It is well established that emotional intelligence is associated with communication skills in medical students’ and residents’ performances in a positive way.

Therefore, emotional intelligence is an important skill that should be incorporated into residents’ or interns’ formal professional skills training (Salovey and Mayer, 1990; Gross and John, 2003; Libbrecht *et al.*, 2014; Bourgeon *et al.*, 2016).

Several studies demonstrated emotional detachment among medical students, in emotionally challenging situations, mostly because of self-preservation which means that students think that they have to distance themselves from the patient’s feelings in order to protect themselves from distress (Doulougeri *et al.*, 2016).

Emotional suppression is used as coping mechanism, and distancing from the patients is also considered a strategy for managing the stressfulness when breaking bad news(Neumann *et al.*, 2011; Burks and Kobus, 2012; Eikeland *et al.*, 2014; Toivonen *et al.*, 2017).

Emotional detachment or loss of feelings was also described in our results. Our findings reveal that residents or interns lack proper coping strategies during challenging patient encounters.

The study findings resonate with those of previous studies dealing with the emotional detachment as a coping strategy. For example, Gaufberg *et al.* reported that medical students described the need to actively suppress emotions in response to the powerful incidents of hospital life (Gaufberg *et al.*, 2010). In medical education, the hidden curriculum may encourage the suppression of emotions and distancing from the patient as an unwritten cultural norm. Several studies report the depersonalization and burnout with the loss of empathy during medical training. (Coulehan and Williams, 2001; Hojat *et al.*, 2004; Coulehan, 2009; Thomas *et al.*, 2007; Neumann *et al.*, 2011; Neufeld and Malin, 2021).

To the best of our knowledge, this is the first study investigating the family medicine residents’ and interns’ emotions during their clinical trainings in Turkey. Our findings suggest a need to further evaluate residents’ or the interns’ emotions during their clinical trainings using ecological momentary assessments (EMAs). EMAs study people’s thoughts and behavior in their daily lives by repeatedly collecting data in an individual’s normal environment, at or close to the time they carry out that behavior. In addition to that, investigations of a larger national sample of residents’ or interns’ emotions will facilitate the understanding of emotions during patient encounters and will help the implementation of reflection or emotion regulation skills programs in Turkey. Conversely, several methodological limitations must be considered when interpreting our findings which has implications on guiding future research. First, this study included only two medical schools of Istanbul with a relatively small number of residents and interns, and second, all data were self-reported and subjective in line with the qualitative studies’ nature. Therefore, the generalizability of our findings may be limited, but they highlight the need for education on emotions (Hamilton-West *et al.*, 2018).

## Conclusion

Emotions are critical in medical education, therefore, residents and interns should be empowered with the skills to acknowledge, accept and regulate them.

Overall, our results have several practice implications. Educators need to understand that challenging encounters evoke many complex emotions in students. Firstly, emotions should be actively discussed in communication skills studies during undergraduate years. Students should be encouraged to accept their emotional experiences and supported in finding strategies for coping with them. Secondly, emotions rising in the authentic clinical education should be systematically reflected. Thirdly, medical teachers need education in reflecting on emotions as part of their teaching practices to be able to constructively address emotional issues. Identifying and normalizing uncomfortable emotions and developing new ways to help learners cope and adapt while remaining empathic and emotionally available to their patients are very important (Toivonen *et al.*, 2017).

Emotions should be explicitly incorporated into medical education, and interns and residents should be supported in coping with these emotions in order to help their professional growth and well-being.
